# Recurrent takotsubo syndrome with worsening of left ventricular outflow obstruction during haemodialysis: a case report

**DOI:** 10.1093/ehjcr/ytaa024

**Published:** 2020-02-21

**Authors:** Takuma Takada, Kentaro Jujo, Issei Ishida, Nobuhisa Hagiwara

**Affiliations:** y1 Department of Cardiology, Tokyo Women’s Medical University, 8-1 Kawada-cho, Shinjuku-ku, Tokyo 162-8666, Japan; y2 Department of Cardiology, Tokyo Women’s Medical University Medical Center East, Tokyo, Japan

**Keywords:** Takotsubo syndrome, Haemodialysis, Left ventricular outflow tract obstruction, Landiolol, Case report

## Abstract

**Background:**

The recurrence rate of takotsubo syndrome (TS) has been reported as 1.8% per patient-year while left ventricular outflow tract (LVOT) obstruction is comorbid in 10–25% of all instances of TS. The clinical course of recurrent TS with associated LVOT while on haemodialysis has rarely been reported.

**Case summary:**

This case report involves a 60-year-old female patient receiving regular haemodialysis who was admitted for chest pain during ballroom dancing. Four years prior, she had suffered TS, and fully recovered after the hospitalization. An emergent coronary angiogram done during the second hospitalization showed no significant stenosis, and left ventriculography demonstrated mid-apical akinesia and basal hyperkinesia. Based on these findings, we diagnosed the recurrence of TS. Later in the admission, chest pain reappeared with the start of haemodialysis. A transthoracic echocardiogram demonstrated mean pressure gradient (PG) of LVOT was 58 mmHg, with systolic anterior motion of the mitral valve and basal-wall hyperkinesia. The main aetiology for her symptoms was considered as an exacerbation of LVOT obstruction due to removing intravascular volume by haemodialysis. After starting landiolol at 3 μg/kg/min, PG of LVOT and symptoms gradually improved with uptitration of landiolol. Finally, her chest pain resolved when mean PG of LVOT was down to 38 mmHg using 10 μg/kg/min of landiolol.

**Discussion:**

To our knowledge, this is the first report of a recurrent TS case comorbid with LVOT obstruction while on regular haemodialysis. Landiolol, the ultrashort-acting beta-blocker, may be a promising therapeutic option for rapid recovery of increased PG due to LVOT obstruction.

**Learning points**

- Recurrent takotsubo syndrome (TS) in a patient with haemodialysis is very rare.
- Landiolol successfully resolved the left ventricular outflow tract obstruction possibly induced by haemodialysis during the acute phase of TS.

## Introduction

Takotsubo syndrome (TS) should be accurately differentiated from acute coronary syndrome to provide appropriate medical management.^1^ Cumulative incidences of recurrent TS is ∼5% at 6 years, and the annual rate of recurrence is only 1–2%.^2^^,^^3^ Further, left ventricular outflow tract (LVOT) obstruction is comorbid in 10–25% of patients with TS and can lead to cardiogenic shock.^4^^,^^5^ However, no formal treatment regimen for patients with TS has yet been established.^6^ Moreover, there were few reports regarding TS with dialysis.^7^ We present the very rare and successful treatment case of a haemodialysis patient who suffered recurrent TS accompanied by LVOT obstruction and cardiogenic shock.

## Timeline

**Table:** 

Time	Events
16 years ago	Haemodialysis was induced by renal sclerosis
1 April 2015	She had suffered takotsubo syndrome (TS). After full recovery, she was followed at the haemodialysis clinic without symptoms.
7 March 2019	She felt chest discomfort during a stressful practice of ballroom dancing.
8 March 2019	She presented to hospital because chest pain was sustained at rest. Electrocardiogram showed that ST-elevations in the inferior and anterior leads with reciprocal changes. The emergent catheterization showed an extensive mid-apical akinesia accompanied by basal hyperkinesia of left ventricle without any significant coronary artery stenoses. We confirmed the diagnosis of recurrent TS.
9 March 2019	She complained chest pain again immediately after starting haemodialysis. A transthoracic echocardiogram (TTE) showed that very high pressure gradient (PG) of left ventricular outflow tract (LVOT) and systolic anterior motion (SAM). After starting landiolol, mean PG of LVOT and symptoms were gradually improving as dose of landiolol was uptitrated.
14 March 2019	A TTE showed that left ventricular asynergy was almost fully recovered, and LVOT obstruction and SAM were no longer documented.
16 March 2019	She was discharged from our hospital without any other complications.
19 July 2019	The patient has been doing well for 4 months after the discharge. Electrocardiogram and TTE indicated normal cardiac functions.

## Case presentation

**Figure 1:**
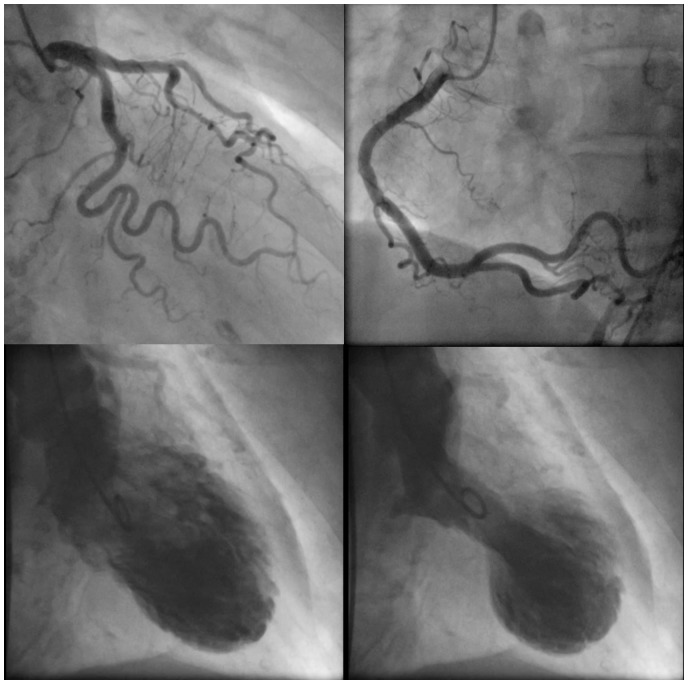
Previous coronary angiography and left ventriculography. *Note*: Coronary artery had no significant stenosis (upper). Left ventriculography of patient was shown in diastolic (lower left) and systolic (lower right) states.

A 60-year-old female patient who had been on the haemodialysis for 16 years due to renal sclerosis was admitted to our hospital for chest pain. She also had medical histories of hypertension, dyslipidaemia, and cured breast cancer. There was no family history of the heart disease. Four years prior to presentation, she had suffered TS; coronary angiography (CAG) and left ventriculography (LVG) at the 1st hospitalization are shown in *Figure 1* and Supplementary material online, *Material S1*. After full recovery from the first episode of TS, she was followed at a haemodialysis clinic without any symptoms of chest pain, with a prescription of 1.25 mg of carvedilol once daily. Prior to the 2nd admission, she felt chest discomfort during a stressful session of ballroom dancing. Since the symptom has remained even at rest for ∼22 h, she presented to the hospital at the next day.

**Figure 2:**
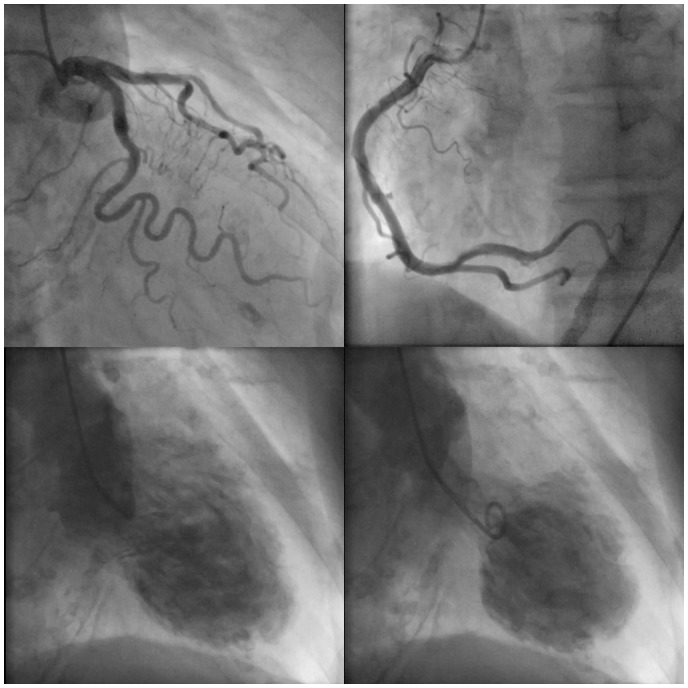
Present coronary angiography and left ventriculography. *Note*: Coronary artery had no significant stenosis (upper). Left ventriculography of patient was shown in diastolic (lower left) and systolic (lower right) states.

**Figure 3:**
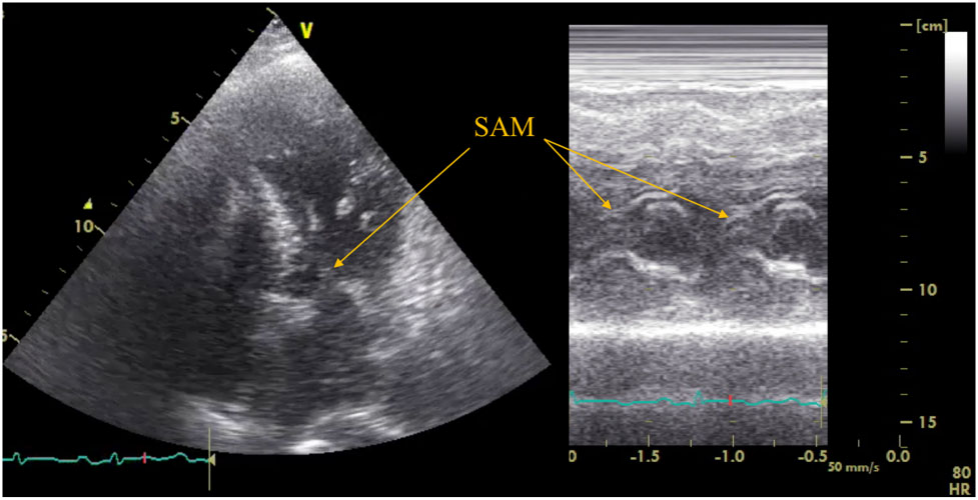
Echocardiography of left ventricular outflow tract obstruction with systolic anterior motion and basal hyperkinesia. LVOT, left ventricular outflow tract; SAM, systolic anterior motion. *Note*: Apical five chamber view (left) and M-mode of SAM in left parasternal long axis view (right) of echocardiography.

**Figure 4:**
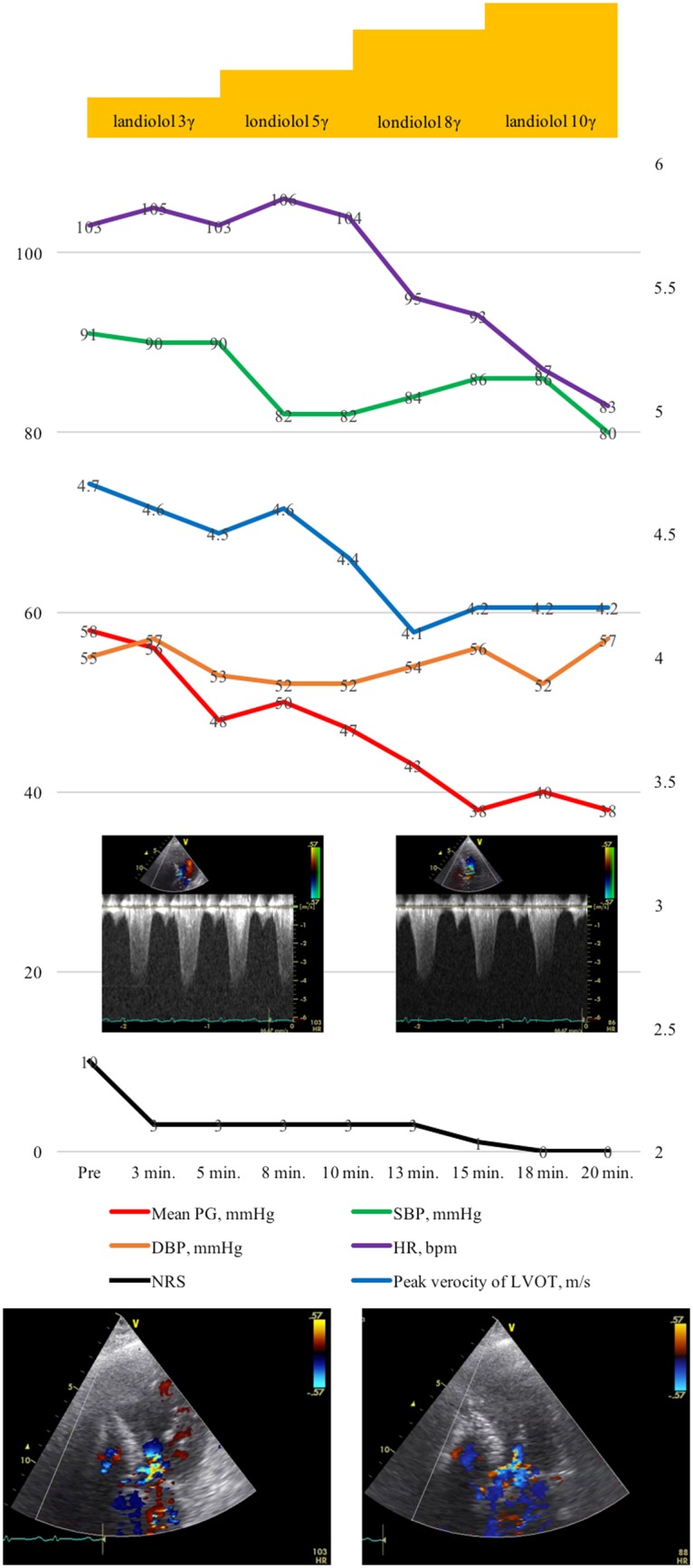
Clinical course of peak velocity and mean PG of LVOT, vital signs and symptoms. DBP, diastolic blood pressure; NRS, numerical rating scale of chest pain; PG, pressure gradient; SBP, systolic blood pressure. *Note*: Apical five chamber view of pre-landiolol (bottom left) and post-landiolol (bottom right) of echocardiography.

At admission, her blood pressure (BP) was 82/58 mmHg and heart rate was 78 beat per minutes with preserved systemic oxygenation. On physical examination, she had a systolic murmur of Levine 2/6 at the second left sternal border, clear lung sounds, and no oedema of lower legs. Electrocardiogram (ECG) showed that sinus rhythm with normal axis, ST-segment elevations in II, III, aVF, and V3–6 leads with ST-segment depressions in I, aVL leads. Laboratory tests showed a haemoglobin level of 13.1 g/dL (within normal limits), creatine kinase-muscle/brain (CK-MB) of 12 U/L (within normal limits), creatine level of 10.1 mg/dL (0.48–0.79 mg/dL), and C-reactive protein level of 0.5 mg/dL (<0.33 mg/dL). A maximum troponin-I level and troponin-T level were 5646.7 pg/mL (<26.2 pg/mL) and 1.460 ng/mL (<0.014 ng/mL), respectively. We also checked the laboratory test of eosinophilic leucocyte ratio, thyroid function, blood sugar, HbA1c, and ferrokinetics and confirmed that these values were within normal limits. A transthoracic echocardiogram (TTE) demonstrated hypokinesia of mid-posterior, inferior, and apical segments of the left ventricle with apical ballooning, sparing the base of each wall, causing a reduced ejection fraction of 40%, accompanied with mild mitral regurgitation (MR). Peak velocity of LVOT was 4.1 m/s. Emergent CAG showed no significant stenosis. Left ventriculography demonstrated an extensive akinesia in both apical and mid portions as well as hyperkinesia in the basal portion of LV with apical ballooning (*Figure 2* and Supplementary material online, *Material S2*). Based on these findings, we confirmed the diagnosis of recurrent TS, and started a standard hospital care including 7 U/kg/h of intravenous unfractionated heparin. The next day, however, she complained of chest pain with haemodialysis although its characteristics were different from initial symptoms. Electrocardiogram did not show any significant changes compared with that on admission. Transthoracic echocardiogram showed that peak systolic velocity and mean pressure gradient (PG) of LVOT were 4.7 m/s and 58 mmHg, respectively. Systolic anterior motion (SAM) of the mitral valve with basal hyperkinesia was also documented (*Figure 3* and Supplementary material online, *Materials S3* and *S4*). Considering that her chest pain was likely due to worsening of LVOT obstruction secondary to decreased intravascular volume from haemodialysis, we started continuous landiolol intravenously at 3 μg/kg/min, and uptitrated it using TTE and vital signs. Her symptoms almost completely resolved when the dose reached at 10 μg/kg/min, shown in *Figure 4*, while peak systolic velocity and mean PG of LVOT were respectively down to 4.2 m/s and 38 mmHg. Then, the dose of landiolol was gradually decreased and continued at 3 μg/kg/min. On Day 3, TTE showed that both mid-apical akinesia and basal hyperkinesia were slightly improved. Peak systolic velocity and mean PG of LVOT were at persistently low levels at 3.1 m/s and 38 mmHg, respectively, with continuous landiolol of 3 μg/kg/min. She was asymptomatic during the 2nd haemodialysis so we switched intravenous landiolol to oral bisoprolol of 1.25 mg. On Day 6, TTE showed that both mid-apical akinesia and basal hyperkinesia were almost recovered, and LVOT obstruction and SAM were no longer noted. Her systolic BP also recovered up to 120 mmHg. She was discharged on Day 8 without any other complications. Discharge medication were 1.25 mg of bisoprolol, 1500 mg of lanthanum carbonate hydrate, 250 mg of ferric citrate hydrate, 10 mg of vonoprazan, 30 mg of domperidone, 0.5 mg of clonazepam, 2 mg of pitavastatin, and 15 mg of nicorandil per day. The radionuclide imaging using iodine-123 meta-iodo-benzyl-guanidine is useful to detect adrenal or ectopic pheochromocytoma in some cases. However, we decided not to perform the radionuclide imaging because the patient in this case did not have any symptoms of pheochromocytoma such as headache, palpitation, and weight loss before the hospitalization and increases of serum catecholamine levels during the index hospitalization. The patient has been doing well for 4 months after the discharge without any symptom or recurrence. Echocardiography at 1 month after discharge showed normal cardiac function. Inverted T wave in I, aVL, and V2–V4 lead of ECG remained for 4 months after discharge, and then totally restored.

## Discussion

The case examines a haemodialysis patient with recurrent TS associated with LVOT obstruction.

Five-year recurrence rates of TS between 5% and 22% have been reported, with the second episode occurring 3 months to 10 years after the first insult.^4^ The patient in this report had suffered the initial TS episode 4 years prior to the recurrence. Although the recurrent TS was of a different anatomical variant in the same patient has been reported,^4^^,^^8^ wall motion abnormalities occurred in the same anatomical LV region in this case. The severity of the second episode was worse than the first because LVOT obstruction had not been observed in the first episode, and her BP on admission was lower during the second episode.

While chronic dialysis patients in Japan continue to increase, up to 329 609 at the end of 2016,^9^ there are only 8 articles including 10 cases of TS with haemodialysis that were found from 3630 articles in PubMed database, indicating that TS does not frequently appear in patients undergoing dialysis.^7^ The causal relationships between TS and dialysis are unclear,^7^ thus our case report can help decision making in these rare occurrences. Although a diagnostic algorithm of TS demonstrated that cardiac magnetic resonance imaging (cMRI) with late gadolinium enhancement was recommended if available after the basic managements,^4^ we did not perform cMRI for this patient, because the patient haemodynamic status was unstable due to LVOT obstruction. Additionally, regular haemodialysis is generally a contraindication for gadolinium enhanced-MRI because of concerning nephrogenic systemic fibrosis (NSF). Schieda *et al*. demonstrated that the risk of developing NSF in patients with acute kidney injury, with severe CKD, and on dialysis was exceedingly low when macrocyclic or newer linear gadolinium-based contrast agents (GBCAs) were used with standard dosages and without short-term repeat injections.^10^ Although these methods are not general in our country, cMRI with macrocyclic, or newer linear GBCA will give the clinical benefits to the patients on haemodialysis in the future.

Haemodynamically significant LVOT obstruction, which was defined as mean PG of LVOT >40 mmHg and systolic BP <110 mmHg, had been observed in 10–25% of TS patients.^4^ Mean PG of LVOT >40 mmHg is one of the risk factors for development of heart failure,^4^ as well as cardiac shock or in-hospital death.^11^ Acute MR is another serious complication, occurring in 14–25% of TS patients,^4^ which may be caused by SAM of the mitral valve with dynamic LVOT obstruction and apical tethering of the subvalvular mitral valve apparatus.^12^ Our case was at high risk of major complications because of the combination of mean PG of LVOT of 58 mmHg with SAM, mild MR, and systolic BP <110 mmHg. Beta-blockers have the effect to lower the PG of LVOT obstruction by reducing basal hypercontractility.^13^ It also protects against stress-induced catecholamine surges.^1^ Landiolol is an intravenous beta-blocker with ultrashort-acting-time and super-high selectivity to beta-1 receptor, which is regarded as a promising option to improve LVOT obstruction. Phenylephrine was another option in the situation of the current case. Phenylephrine has a benefit of raising BP and can preserve haemodynamic state, but we have to be careful to the complication which is the subsequent reflex bradycardia. As an alternative, we determined landiolol as the first medication in this case because of its short-acting time and feasibility for dose-titration. However, we considered that phenylephrine could be added on the treatment if the haemodynamic state remained unstable.

As far as we investigated, there were only three articles reporting the effectiveness of landiolol on LVOT obstruction.^13–15^ Moreover, there has been no publication evaluating the impact of landiolol in the setting of haemodialysis. Therefore, we concluded that this case was valuable as the first report of recurrent TS in a haemodialysis patient. We also suggested that ultrashort-acting intravenous beta-blockers were one of the promising options for TS with the worsening LVOT obstruction because of its feasibility for dose-titration under real-time monitoring.

## Conclusions

This may be the first case report of recurrent TS with haemodialysis and LVOT obstruction, with successful treatment of intravenous landiolol. However, the effect of landiolol for TS was not evaluated by randomized controlled trial, and further investigations are needed to find the best way to use this drug in high-risk TS patients.

## Lead author biography

**Figure:**
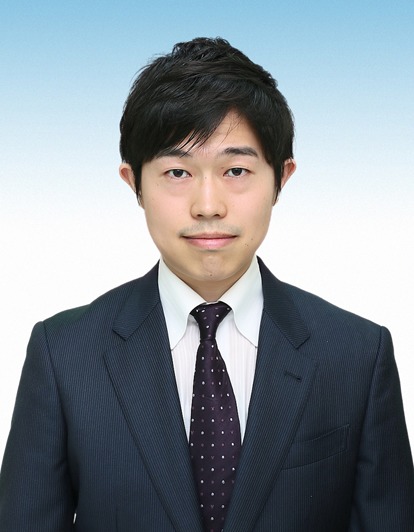


Takuma Takada, MD, is PhD trainee at Department of Cardiology, Tokyo Women’s Medical University from 2019, working at CCU. He was graduated from Osaka Medical College in 2012. He finished resident program (2012–14) and fellowship in Cardiology (2014–19) in Japan. Membership: Japanese Circulation Society, Japanese Association of Cardiovascular Intervention and Therapeutics.

## Supplementary material

Supplementary material is available at *European Heart Journal - Case Reports* online.

## Supplementary Material

ytaa024_Supplementary_Data
