# Supplementary material for: Recurrent takotsubo syndrome with worsening of left ventricular outflow obstruction during haemodialysis: a case report
Source: Eur Heart J Case Rep. 2020 Feb 21;4(2):1–6. doi: 10.1093/ehjcr/ytaa024 (PMC7180539; doi:10.1093/ehjcr/ytaa024)
Supplement: ytaa024_Supplementary_Data [file ytaa024_supplementary_data.zip › ytaa024-Suppl_Data/EHJ-CR_Slide_Set.191207.pptx]

## Slide 1
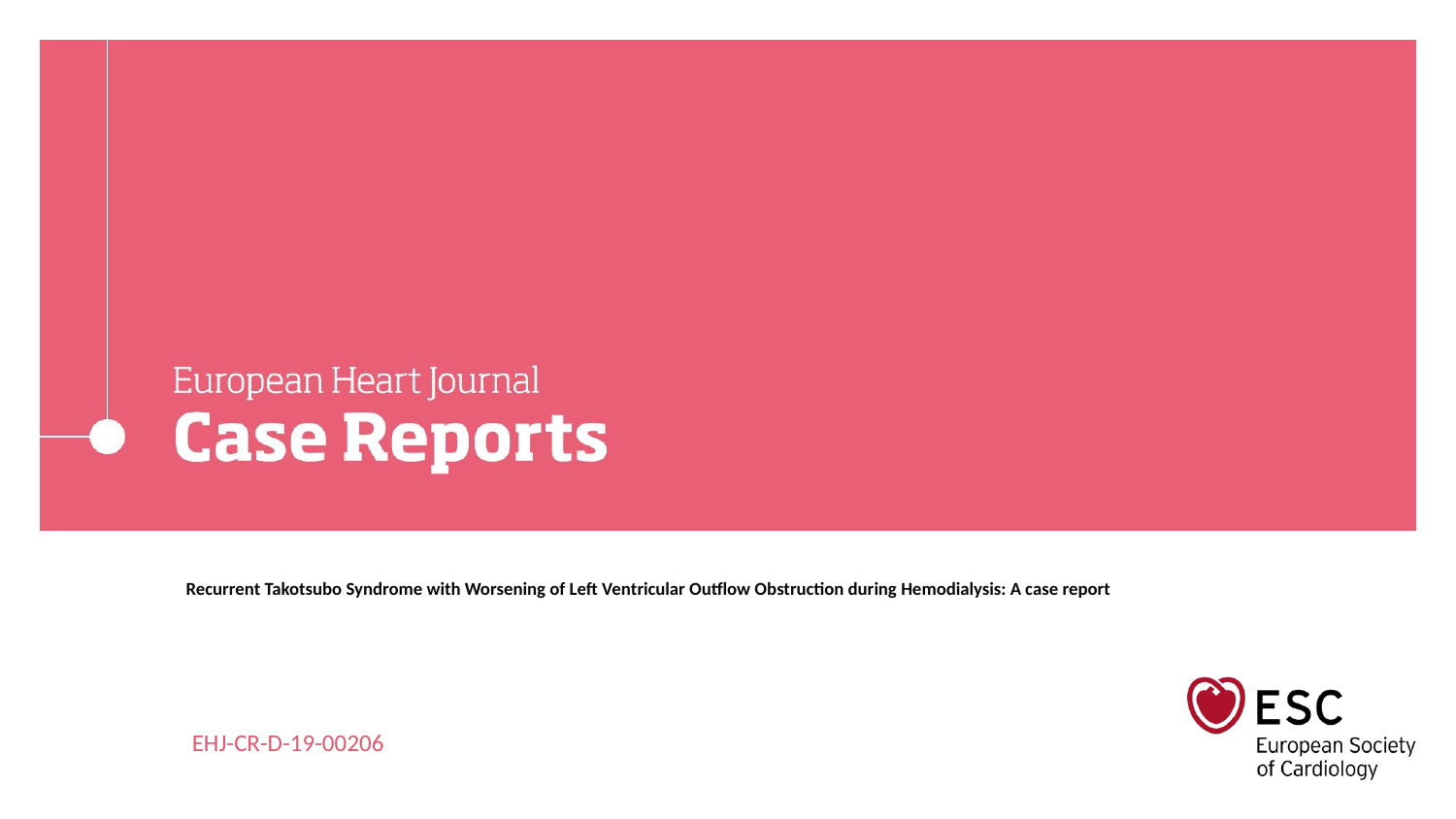

# Recurrent Takotsubo Syndrome with Worsening of Left Ventricular Outflow Obstruction during Hemodialysis: A case report
EHJ-CR-D-19-00206

## Slide 2
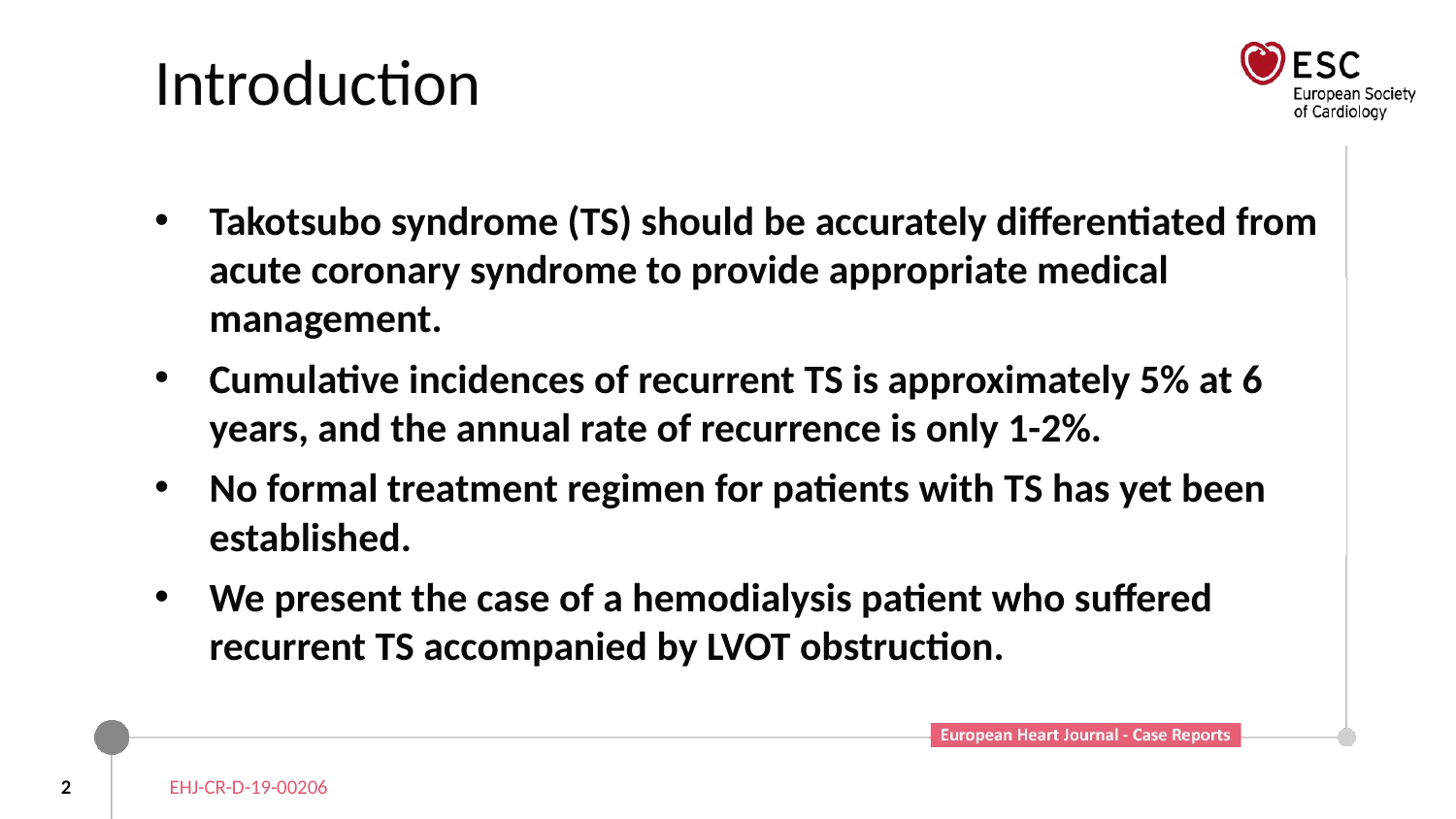

# Introduction
Takotsubo syndrome (TS) should be accurately differentiated from acute coronary syndrome to provide appropriate medical management.
Cumulative incidences of recurrent TS is approximately 5% at 6 years, and the annual rate of recurrence is only 1-2%.
No formal treatment regimen for patients with TS has yet been established.
We present the case of a hemodialysis patient who suffered recurrent TS accompanied by LVOT obstruction.
2
EHJ-CR-D-19-00206

## Slide 3
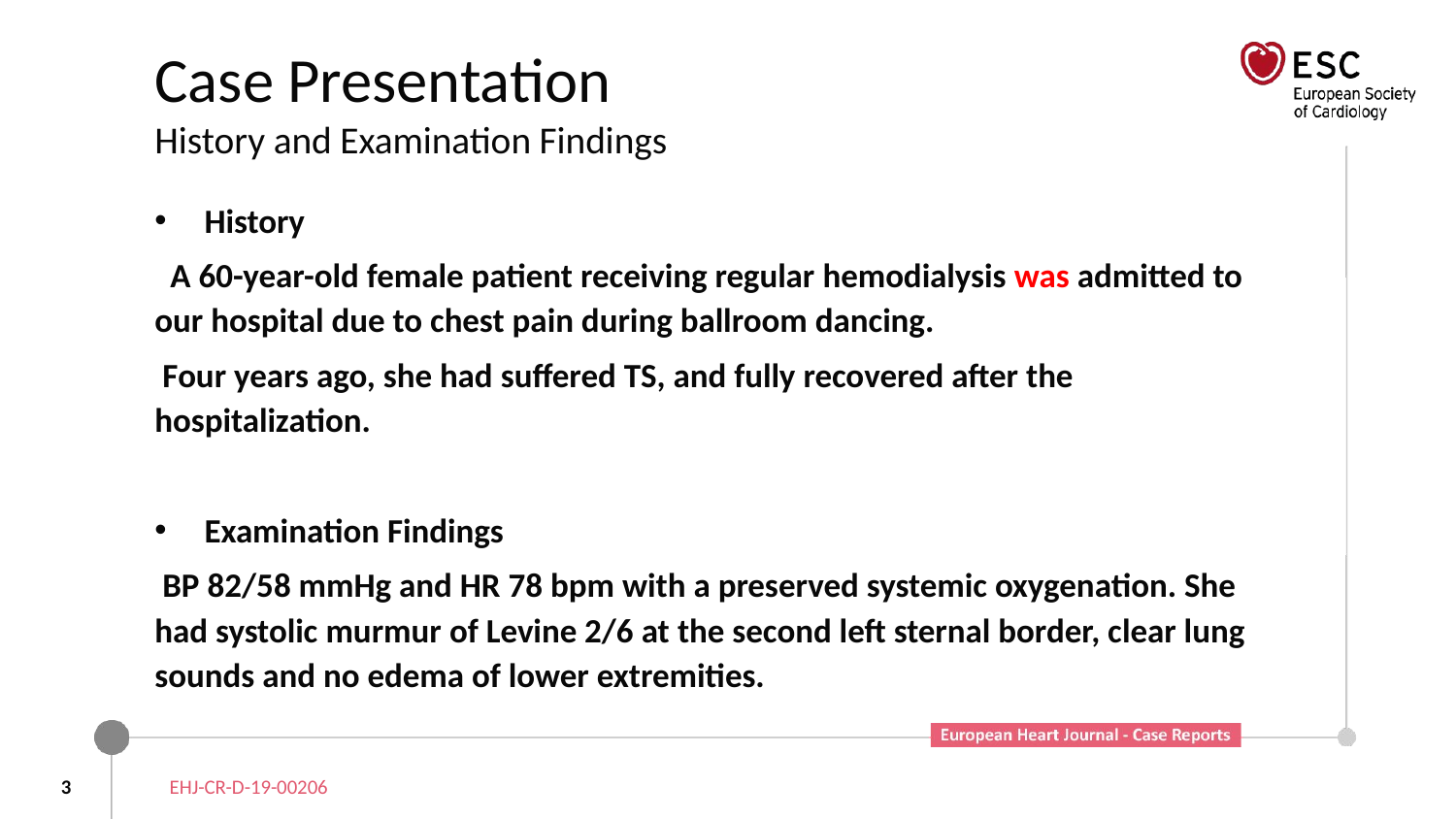

# Case Presentation History and Examination Findings
History
 A 60-year-old female patient receiving regular hemodialysis was admitted to our hospital due to chest pain during ballroom dancing.
 Four years ago, she had suffered TS, and fully recovered after the hospitalization.
Examination Findings
 BP 82/58 mmHg and HR 78 bpm with a preserved systemic oxygenation. She had systolic murmur of Levine 2/6 at the second left sternal border, clear lung sounds and no edema of lower extremities.
3
EHJ-CR-D-19-00206

## Slide 4
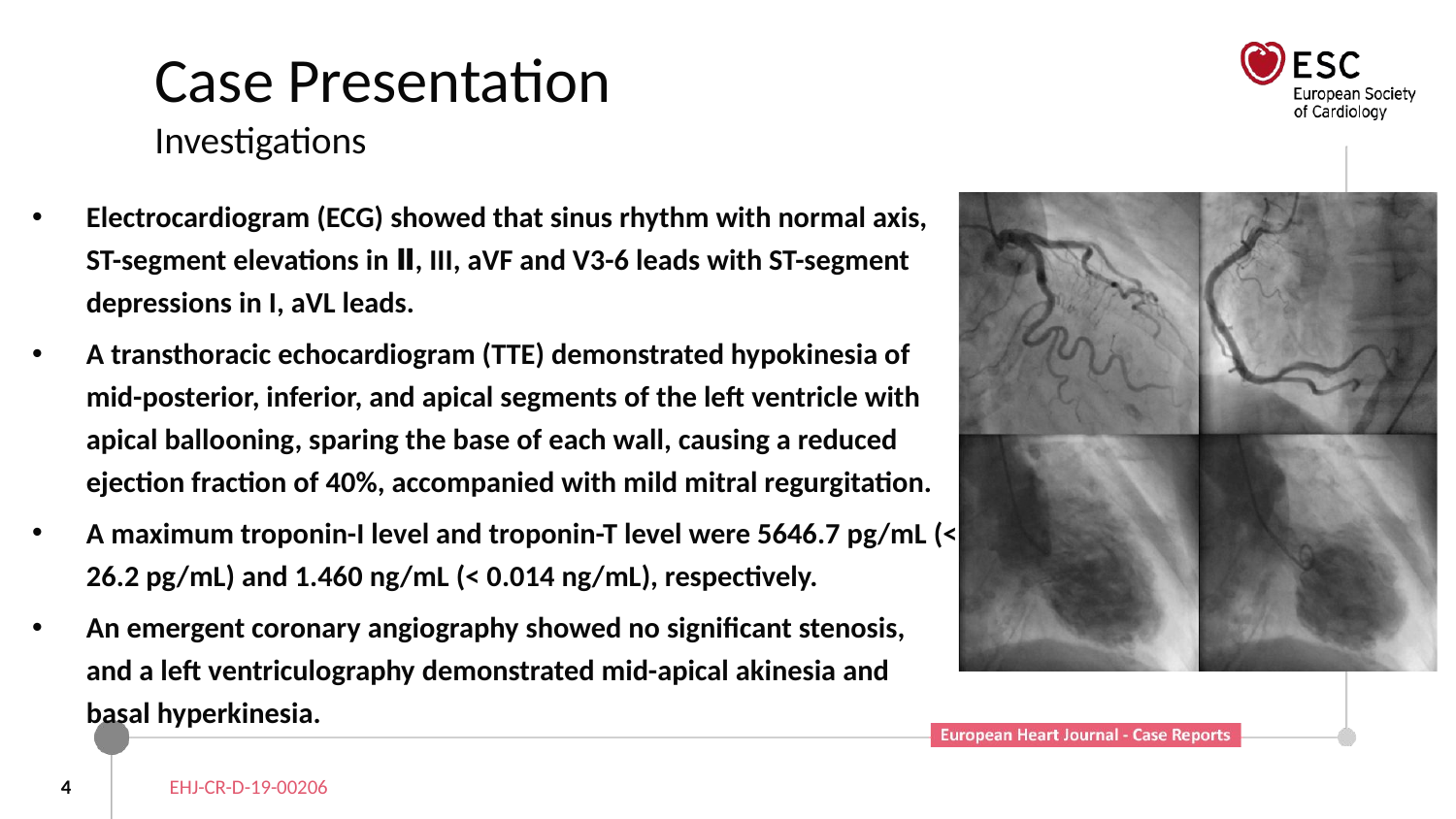

# Case Presentation Investigations
Electrocardiogram (ECG) showed that sinus rhythm with normal axis, ST-segment elevations in Ⅱ, III, aVF and V3-6 leads with ST-segment depressions in I, aVL leads.
A transthoracic echocardiogram (TTE) demonstrated hypokinesia of mid-posterior, inferior, and apical segments of the left ventricle with apical ballooning, sparing the base of each wall, causing a reduced ejection fraction of 40%, accompanied with mild mitral regurgitation.
A maximum troponin-I level and troponin-T level were 5646.7 pg/mL (< 26.2 pg/mL) and 1.460 ng/mL (< 0.014 ng/mL), respectively.
An emergent coronary angiography showed no significant stenosis, and a left ventriculography demonstrated mid-apical akinesia and basal hyperkinesia.
4
EHJ-CR-D-19-00206

## Slide 5
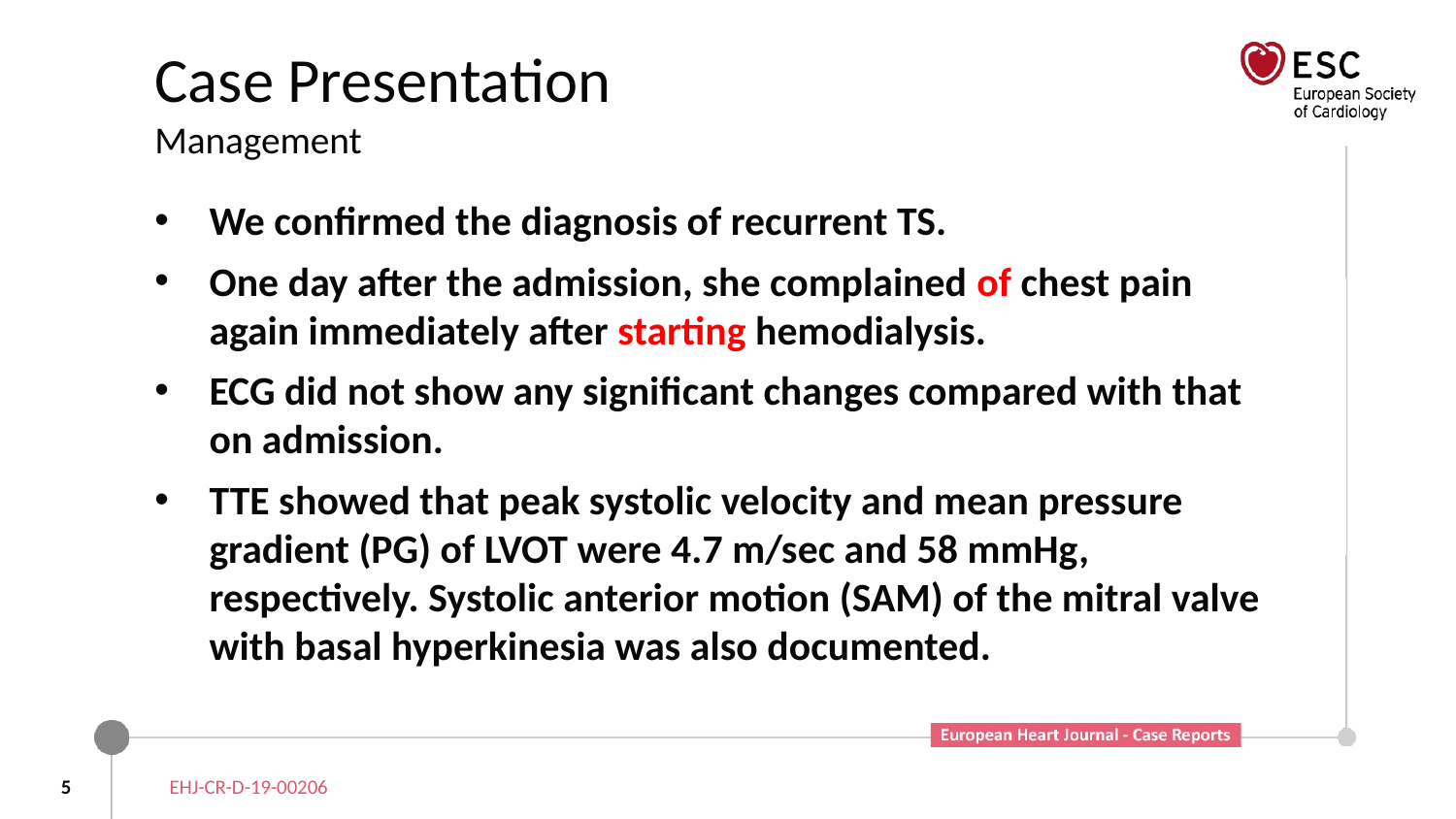

# Case Presentation Management
We confirmed the diagnosis of recurrent TS.
One day after the admission, she complained of chest pain again immediately after starting hemodialysis.
ECG did not show any significant changes compared with that on admission.
TTE showed that peak systolic velocity and mean pressure gradient (PG) of LVOT were 4.7 m/sec and 58 mmHg, respectively. Systolic anterior motion (SAM) of the mitral valve with basal hyperkinesia was also documented.
5
EHJ-CR-D-19-00206

## Slide 6
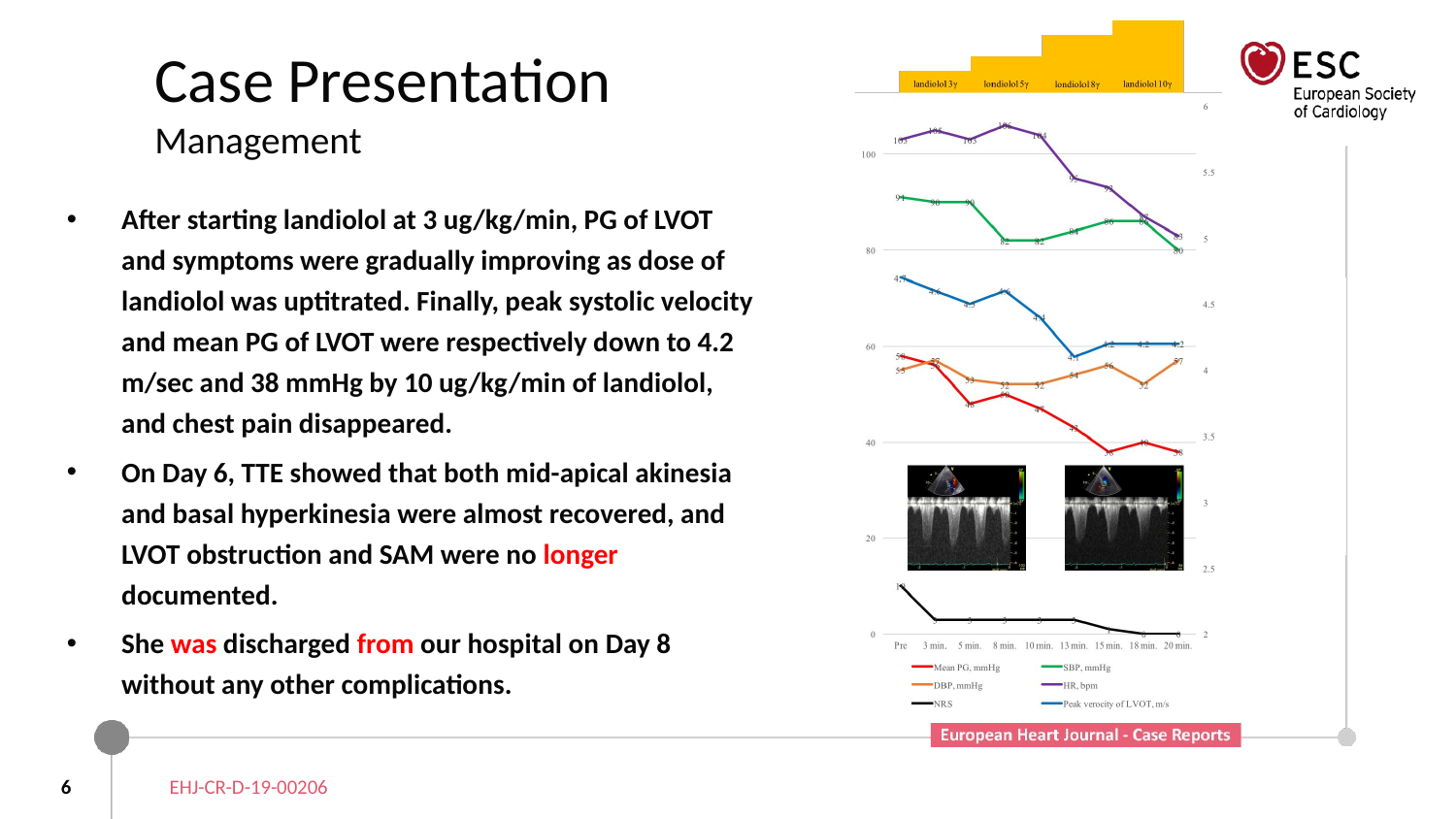

# Case Presentation Management
After starting landiolol at 3 ug/kg/min, PG of LVOT and symptoms were gradually improving as dose of landiolol was uptitrated. Finally, peak systolic velocity and mean PG of LVOT were respectively down to 4.2 m/sec and 38 mmHg by 10 ug/kg/min of landiolol, and chest pain disappeared.
On Day 6, TTE showed that both mid-apical akinesia and basal hyperkinesia were almost recovered, and LVOT obstruction and SAM were no longer documented.
She was discharged from our hospital on Day 8 without any other complications.
6
EHJ-CR-D-19-00206

## Slide 7
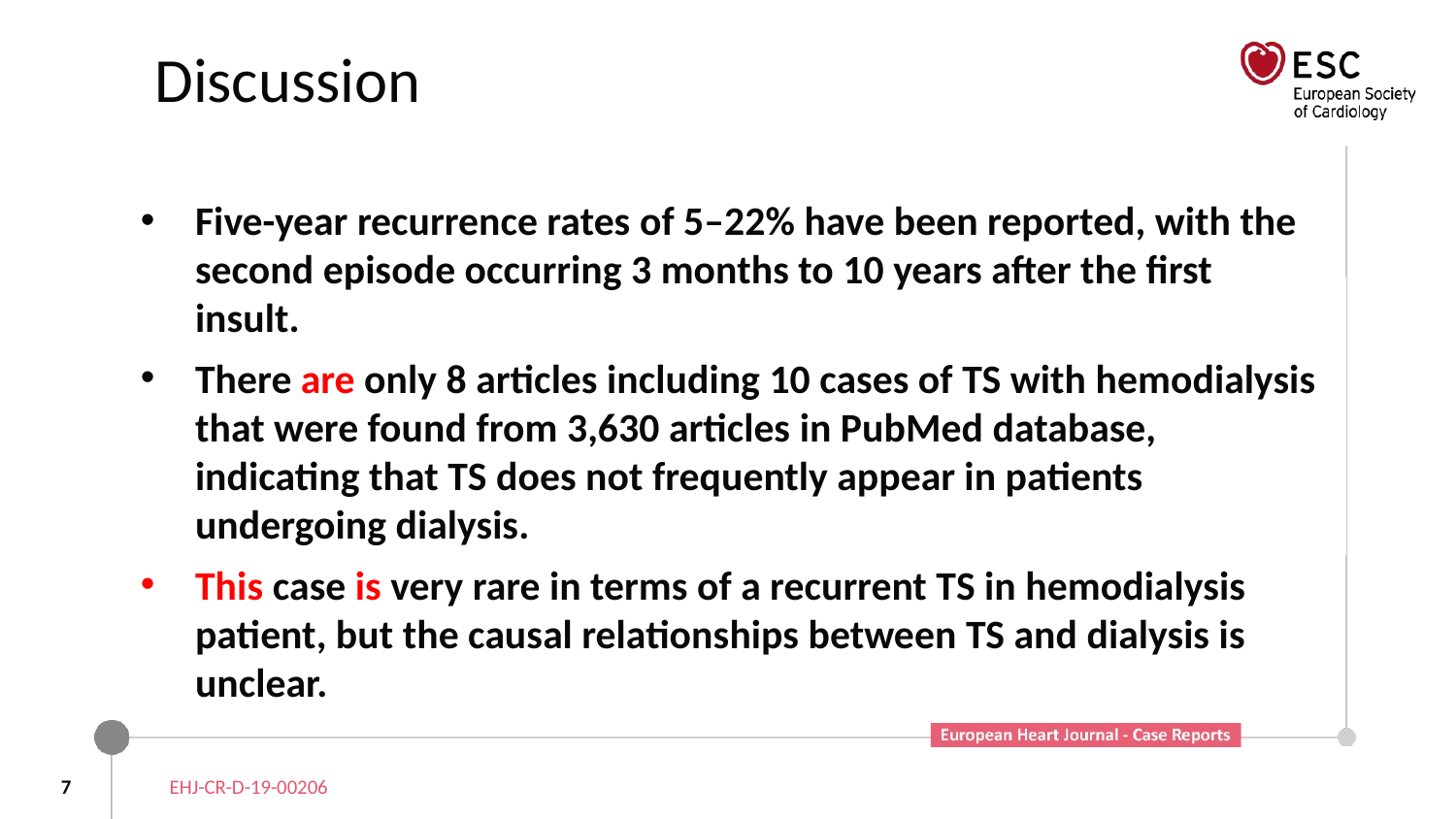

# Discussion
Five-year recurrence rates of 5–22% have been reported, with the second episode occurring 3 months to 10 years after the first insult.
There are only 8 articles including 10 cases of TS with hemodialysis that were found from 3,630 articles in PubMed database, indicating that TS does not frequently appear in patients undergoing dialysis.
This case is very rare in terms of a recurrent TS in hemodialysis patient, but the causal relationships between TS and dialysis is unclear.
7
EHJ-CR-D-19-00206

## Slide 8
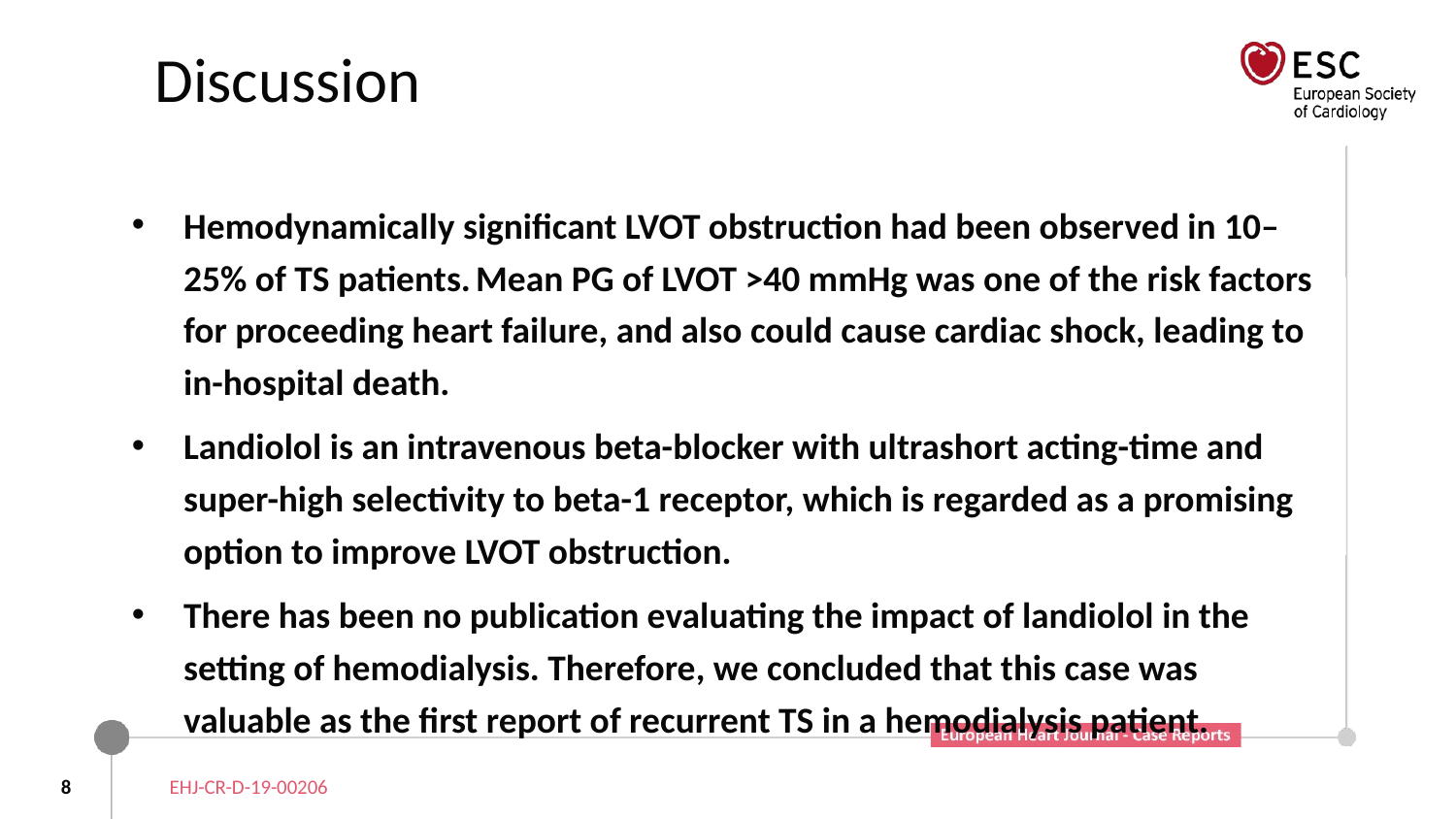

# Discussion
Hemodynamically significant LVOT obstruction had been observed in 10–25% of TS patients. Mean PG of LVOT >40 mmHg was one of the risk factors for proceeding heart failure, and also could cause cardiac shock, leading to in-hospital death.
Landiolol is an intravenous beta-blocker with ultrashort acting-time and super-high selectivity to beta-1 receptor, which is regarded as a promising option to improve LVOT obstruction.
There has been no publication evaluating the impact of landiolol in the setting of hemodialysis. Therefore, we concluded that this case was valuable as the first report of recurrent TS in a hemodialysis patient.
8
EHJ-CR-D-19-00206

## Slide 9
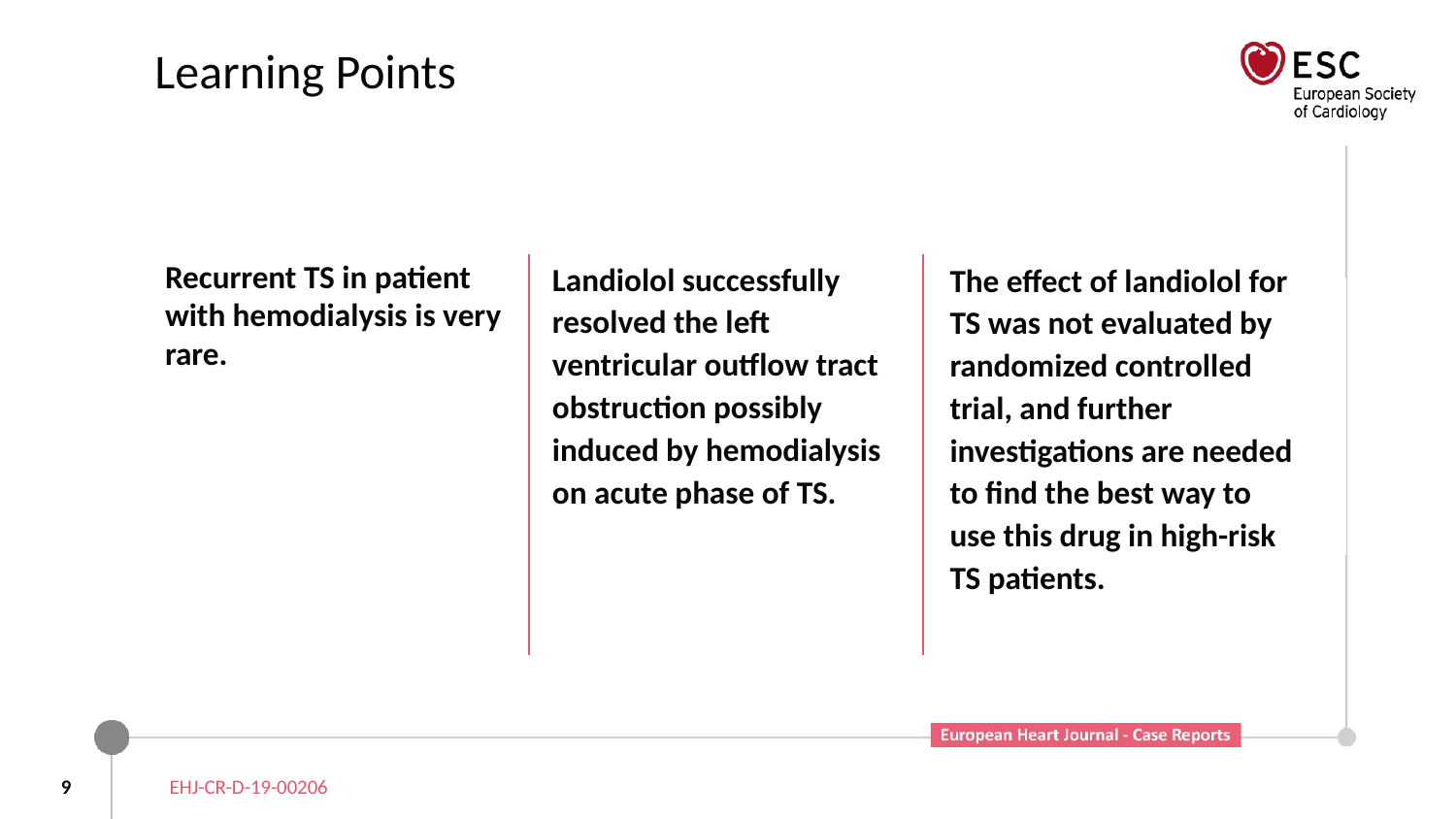

# Learning Points
Landiolol successfully resolved the left ventricular outflow tract obstruction possibly induced by hemodialysis on acute phase of TS.
The effect of landiolol for TS was not evaluated by randomized controlled trial, and further investigations are needed to find the best way to use this drug in high-risk TS patients.
Recurrent TS in patient with hemodialysis is very rare.
9
EHJ-CR-D-19-00206

## Slide 10
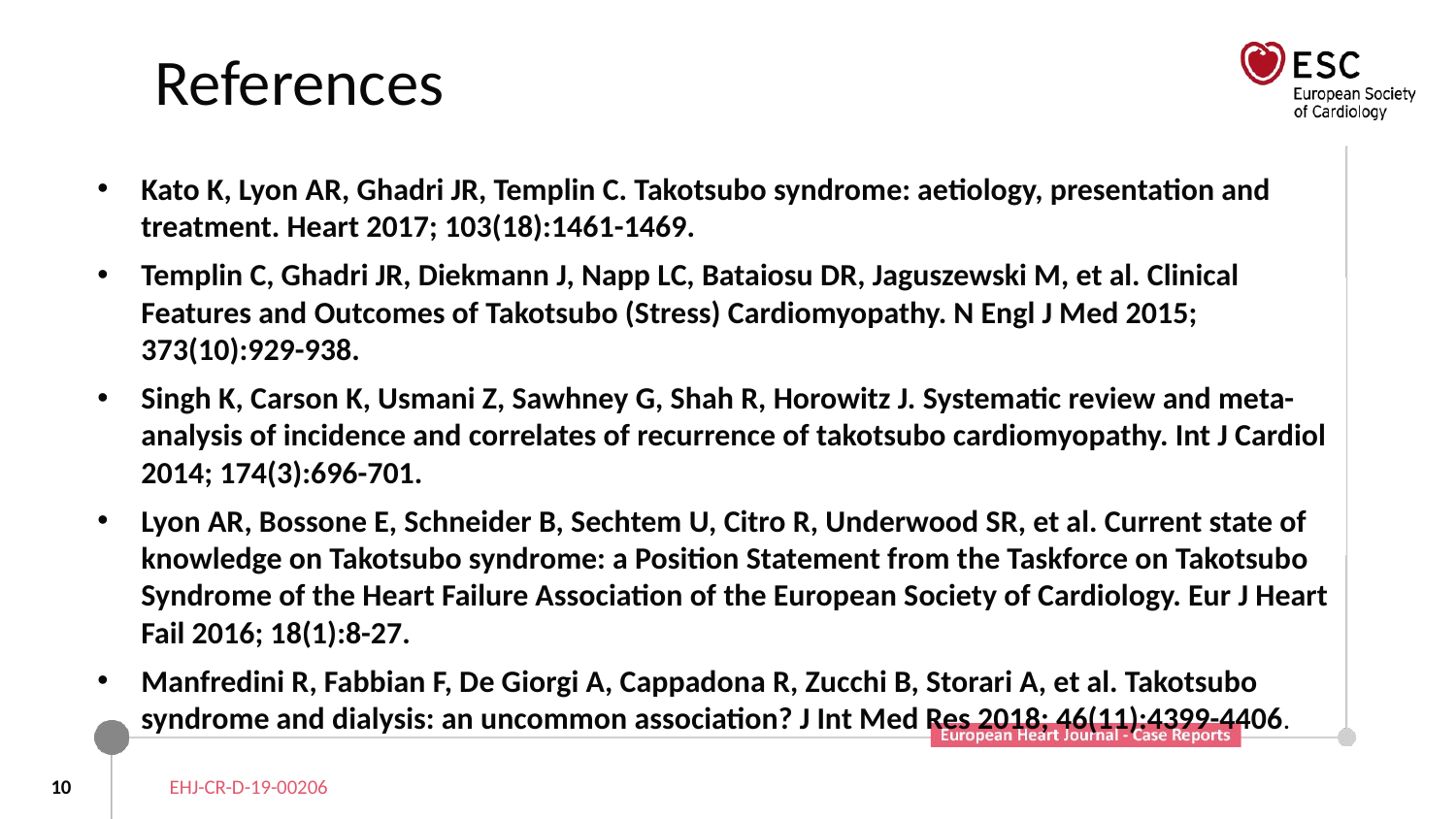

# References
Kato K, Lyon AR, Ghadri JR, Templin C. Takotsubo syndrome: aetiology, presentation and treatment. Heart 2017; 103(18):1461-1469.
Templin C, Ghadri JR, Diekmann J, Napp LC, Bataiosu DR, Jaguszewski M, et al. Clinical Features and Outcomes of Takotsubo (Stress) Cardiomyopathy. N Engl J Med 2015; 373(10):929-938.
Singh K, Carson K, Usmani Z, Sawhney G, Shah R, Horowitz J. Systematic review and meta-analysis of incidence and correlates of recurrence of takotsubo cardiomyopathy. Int J Cardiol 2014; 174(3):696-701.
Lyon AR, Bossone E, Schneider B, Sechtem U, Citro R, Underwood SR, et al. Current state of knowledge on Takotsubo syndrome: a Position Statement from the Taskforce on Takotsubo Syndrome of the Heart Failure Association of the European Society of Cardiology. Eur J Heart Fail 2016; 18(1):8-27.
Manfredini R, Fabbian F, De Giorgi A, Cappadona R, Zucchi B, Storari A, et al. Takotsubo syndrome and dialysis: an uncommon association? J Int Med Res 2018; 46(11):4399-4406.
10
EHJ-CR-D-19-00206
